# Personal and Parental Weight Misperception and Self-Reported Attempted Weight Loss in US Children and Adolescents, National Health and Nutrition Examination Survey, 2007–2008 and 2009–2010

**DOI:** 10.5888/pcd11.140123

**Published:** 2014-07-31

**Authors:** Han-Yang Chen, Stephenie C. Lemon, Sherry L. Pagoto, Bruce A. Barton, Kate L. Lapane, Robert J. Goldberg

**Affiliations:** Author Affiliations: Stephenie C. Lemon, Sherry L. Pagoto, Bruce A. Barton, Kate L. Lapane, Robert J. Goldberg, University of Massachusetts Medical School, Worcester, Massachusetts.

## Abstract

**Introduction:**

The objective of our study was to describe perceptions of child weight status among US children, adolescents, and their parents and to examine the extent to which accurate personal and parental perception of weight status is associated with self-reported attempted weight loss.

**Methods:**

Our study sample comprised 2,613 participants aged 8 to 15 years in the National Health and Nutrition Examination Survey from the 2 most recent consecutive cycles (2007–2008 and 2009–2010). Categories of weight perception were developed by comparing measured to perceived weight status. Multivariable logistic regression analyses were used to examine the association between weight misperception and self-reported attempted weight loss.

**Results:**

Among children and adolescents, 27.3% underestimated and 2.8% overestimated their weight status. Among parents, 25.2% underestimated and 1.1% overestimated their child’s weight status. Logistic regression analyses showed that the odds of self-reported attempted weight loss was 9.5 times as high (95% confidence interval [CI]: 3.8–23.6) among healthy-weight children and adolescents who overestimated their weight status as among those who perceived their weight status accurately; the odds of self-reported attempted weight loss were 3.9 (95% CI, 2.4–6.4) and 2.9 (95% CI, 1.8–4.6) times as high among overweight and obese children and adolescents, respectively, who accurately perceived their weight status than among those who underestimated their weight status. Parental misperception of weight was not significantly associated with self-reported attempted weight loss among children and adolescents who were overweight or obese.

**Conclusion:**

Efforts to prevent childhood obesity should incorporate education for both children and parents regarding the proper identification and interpretation of actual weight status. Interventions for appropriate weight loss can target children directly because one of the major driving forces to lose weight comes from the child’s perception of his or her weight status.

## MEDSCAPE CME

Medscape, LLC is pleased to provide online continuing medical education (CME) for this journal article, allowing clinicians the opportunity to earn CME credit.

This activity has been planned and implemented in accordance with the Essential Areas and policies of the Accreditation Council for Continuing Medical Education through the joint sponsorship of Medscape, LLC and Preventing Chronic Disease. Medscape, LLC is accredited by the ACCME to provide continuing medical education for physicians.

Medscape, LLC designates this Journal-based CME activity for a maximum of 1 **AMA PRA Category 1 Credit(s)™**. Physicians should claim only the credit commensurate with the extent of their participation in the activity.

All other clinicians completing this activity will be issued a certificate of participation. To participate in this journal CME activity: (1) review the learning objectives and author disclosures; (2) study the education content; (3) take the post-test with a 75% minimum passing score and complete the evaluation at www.medscape.org/journal/pcd (4) view/print certificate.

**Release date: July 31, 2014; Expiration date: July 31, 2015**

### Learning Objectives

Upon completion of this activity, participants will be able to:

- Analyze the problem of pediatric obesity in the United States
- Assess misperceptions of weight status among children and adolescents
- Evaluate how misperceptions of weight status may be linked with weight loss behaviors
- Distinguish additional factors associated with more weight loss behaviors

**EDITORS**

Rosemarie Perrin, editor, *Preventing Chronic Disease*. Disclosure: Rosemarie Perrin has disclosed no relevant financial relationships.

**CME AUTHOR**

Charles P. Vega, MD, Associate Professor and Residency Director, Department of Family Medicine, University of California, Irvine. Disclosure: Charles P. Vega, MD, has disclosed the following relevant financial relationships: Served as an advisor or consultant for: McNeil Pharmaceuticals.

**AUTHORS AND CREDENTIALS**

Disclosures: Han-Yang Chen, MS; Stephenie C. Lemon, PhD; Sherry L. Pagoto, PhD; Bruce A. Barton, PhD; Kate L. Lapane, PhD; and Robert J. Goldberg, PhD have disclosed no relevant financial relationships.

Affiliations: Han-Yang Chen, Stephenie C. Lemon, Sherry L. Pagoto, Bruce A. Barton, Kate L. Lapane, Robert J. Goldberg, University of Massachusetts Medical School, Worcester, Massachusetts.

## Introduction

The prevalence of obesity has more than doubled among US children and tripled among US adolescents during the past 30 years (1,2). Data from the National Health and Nutrition Examination Survey (NHANES) 2009–2010 showed that nearly one-third of children and adolescents living in the United States were overweight or obese (1).Obese children and adolescents are at increased risk for short-term health consequences (3), obesity in adulthood (4), and health problems in adulthood (5).

As the Health Belief Model suggests, to improve health, people need to be aware of the severity of their condition and acknowledge the benefits of taking steps to make positive changes in their health behavior (6–8). In childhood obesity prevention and reduction, children’s self-perceived weight status as well as parents’ perception of their children’s weight status may be important, because parents have considerable control over their children’s lives, including opportunities to offer obesity interventions (9,10). However, weight misperception, the discordance between an individual’s actual versus perceived weight status, has repeatedly been documented among overweight and obese people. Prior studies suggest that children and their parents often underestimate the child’s weight status (11–13).

Despite misperceptions of children’s weight status, few studies using nationally representative samples have examined the association between weight misperception among children, adolescents, or their parents and attempts at weight loss. Accurate weight perception would seem necessary to identify risks for obesity-related chronic diseases and health behaviors, and recognition of overweight status may motivate overweight children and adolescents to modify their adverse lifestyle practices.

The objective of this study was to describe estimates of weight misperception among a nationally representative sample of US children and adolescents aged 8 to 15 years and their parents. We also examined the extent to which accurate personal and parental weight perception was associated with self-reported attempted weight loss by children and adolescents. Data from the 2007–2008 and 2009–2010 National Health and Nutrition Examination Survey (NHANES) were used for this investigation.

## Methods

### Study population and design

We examined data from NHANES, a nationally representative and multistage probability sample of the civilian, noninstitutionalized US population conducted by the National Center for Health Statistics (14,15). NHANES participants in these 2 surveys were interviewed in their homes on various demographic and health-related topics by using the computer-assisted personal interview (CAPI) system. For survey participants under 16 years of age, a proxy (parent) provided information, including child’s age, sex, race/ethnicity, health insurance status, and annual household income. The proxy was also asked about his or her perception of the child’s weight status. In addition, children and adolescents underwent a thorough physical examination, including measured height and weight using a standard protocol (16) in a mobile examination center (MEC). Self-reported weight history questions were asked directly for participants aged 8 to 15 years as part of the MEC interview.

Since 1999, NHANES data have been released biennially. We combined data from the 2 most recent consecutive cycles (2007–2008 and 2009–2010) to increase the available sample size. The overall unweighted NHANES response rates for the interview samples of these 2 surveys were 78.4% (2007–2008) and 79.4% (2009–2010); for the examined samples these rates were 75.4% (2007–2008) and 77.3% (2009–2010). Our study population included participants aged 8 to 15 years who had proxy-reported home interviews and who were examined and interviewed at MECs. Participants without valid information on weight status and self-reported attempted weight loss were excluded (n = 202). We also excluded participants who were clinically underweight (n = 80), because no participant in this group reported trying to lose weight. This resulted in a final sample study size of 2,613 participants. We did not seek ethics approval from our institutional review board because the NHANES data are completely de-identified and publicly available.

### Exposure variables

We used 3 weight-related measures, including personal and parental weight perception and measured weight status, to operationalize the primary exposure variables: personal weight misperception and parental weight misperception.

#### Perception of weight status from child and parent

We used CAPI questionnaires to measure personal weight perception by asking children and adolescents, “Do you consider yourself now to be . . . ?” Response options were “fat or overweight,” “too thin,” and “about the right weight.” To assess parental perception of child’s weight status, during the home interview parents were asked the question, “Do you consider (child or adolescent) now to be . . . ?” Response options were “overweight,” “underweight,” and “about the right weight.”

#### Measured weight status

According to age- and sex-specific percentiles for body mass index (BMI) from the Centers for Disease Control and Prevention (CDC) (17), participants’ weight status was calculated by using measured height and weight obtained by trained interviewers during the physical examinations and proxy-reported age and sex obtained during the in-home interviews. Percentile categories of BMI were assigned according to the 2000 CDC growth charts (≥95th percentile, obese; 85th to <95th percentile, overweight; 5th to <85th percentile, healthy weight; and <5th percentile, underweight) (17).

#### Misperception of weight status

We constructed 2 exposure variables of personal and parental weight misperception on the basis of perceived and measured weight status.

Children were determined to have overestimated or underestimated their weight status if they perceived their weight status as being heavier or lighter than their measured weight status. Children whose self-perceived weight status corresponded with their measured weight status were classified as accurate perceivers. Thus, personal weight misperception was categorized as overestimated, accurate, or underestimated. Following the same criteria, parental weight misperception was also categorized into the same 3 strata: overestimated, accurate, or underestimated.

### Outcome variable

The primary outcome variable was self-reported attempted weight loss by children and adolescents. This was assessed by asking children and adolescents, “Which of the following are you trying to do about your weight?” Possible responses were “lose weight,” “gain weight,” “stay the same weight,” and “not trying to do anything about your weight.” Since self-reported attempted weight loss was defined as “trying to lose weight,” we collapsed response options into 2 categories as either 1) trying to lose weight or 2) not trying to lose weight (includes “gain weight,” “stay the same weight,” and “not trying to do anything about your weight”).

### Covariates

Several covariates associated with weight loss in prior studies (13,18) were examined, including a child’s proxy-reported age (8–15 years), sex (male, female), race/ethnicity (non-Hispanic white, non-Hispanic black, Hispanic, or non-Hispanic other race), health insurance status (yes, no), and annual household income (<$20,000, $20,000–<$35,000, $35,000–<$55,000, $55,000–<$75,000, ≥$75,000).

### Data analysis

Prevalence estimates of personal and parental misperception of the child’s weight were calculated with frequency distributions. Differences in sociodemographic characteristics according to self-reported attempted weight loss were examined by using the χ^2^ test for categorical variables and the *t* test for continuous variables; these analyses were stratified by the child’s BMI classification.

Multivariable logistic regression analyses were used to examine the association between personal weight misperception, parental weight misperception, and self-reported attempted weight loss among children and adolescents according to their BMI classification strata. These analyses adjusted for several potential confounding variables including the child’s age, sex, race/ethnicity, health insurance status, and annual household income. Interaction of personal or parental weight misperception with sex or race/ethnicity, and interaction between personal and parental weight misperception were examined. We estimated adjusted odds ratios (ORs) and 95% confidence intervals (CIs); sample weights and the stratification and clustering of the NHANES design were taken into account in all analyses.

## Results

### Sociodemographic characteristics of study population

A total of 2,613 participants (weighted N = 29,801,979) aged 8 to 15 years were included in the final sample (Table 1). On the basis of measured BMI, 62.7% of children and adolescents were of healthy weight, 17.4% were overweight, and 19.8% were obese. Overall, the average age of our sample was 11.6 years (standard deviation, 2.3 y), 50.4% were aged 12 to 15 years, 49.1% were female, and 58.2% were white. The majority had health insurance coverage (90.4%) and an annual household income at or above $20,000 (83.9%). About one-third (33.4%) of children and adolescents self-reported attempted weight loss, including 15.2%, 49.0%, and 77.5% of healthy weight, overweight, and obese groups, respectively. No significant differences were found in the distribution of examined variables between trying to lose weight and not trying to lose weight, except for sex in the healthy weight group and race/ethnicity in the overweight and obese groups.

**Table 1:** Sample Characteristics by Active Weight Control by Healthy Weight, Overweight, and Obese Children and Adolescents Aged 8 to 15 Years in the United States (N = 2,613), 2007–2010^a^

Child and Adolescent Characteristic	Healthy Weight (n = 1,557)		*P* Value	Overweight (n = 461)		*P* Value	Obese (n = 595)		*P* Value
	Trying to Lose Weight (n = 252)	Not Trying to Lose Weight (n = 1,305)		Trying to Lose Weight (n = 248)	Not Trying to Lose Weight (n = 213)		Trying to Lose Weight (n = 477)	Not Trying to Lose Weight (n = 118)	
**Age, years, mean (SD)**	12.0 (2.3)	11.6 (2.3)	.11	11.8 (2.4)	11.4 (2.2)	.20	11.4 (2.3)	11.4 (2.3)	.99
**Race/ethnicity, %**									
Non-Hispanic white	57.2	62.0	.39	50.8	61.8	.001	47.3	59.4	.019
Non-Hispanic black	12.9	12.3		16.4	11.5		19.1	13.8	
Hispanic	22.1	17.3		29.3	16.5		28.2	19.4	
Non-Hispanic other	7.8	8.3		3.5	10.3		5.5	7.4	
**Female, %**	63.2	46.3	<.001	55.0	52.8	.72	47.4	38.9	.11
**Had health insurance, %**	89.7	91.3	.45	83.5	90.8	.05	89.9	94.3	.14
**Annual household income ($), %**									
<20,000	17.7	13.1	.47	19.8	15.2	.74	22.4	22.0	.07
20,000–<35,000	16.0	18.5		17.7	15.4		26.6	16.4	
35,000–<55,000	12.6	14.4		13.4	13.5		15.4	12.1	
55,000–<75,000	16.6	13.9		21.2	22.9		11.1	9.7	
≥75,000	37.1	40.1		27.9	33.0		24.5	39.9	

Abbreviation: SD, standard deviation.

a Weighted N = 29,801,979; values are expressed as weighted mean or percentages.

### Personal and parental perceived weight status

Among all children and adolescents in our sample, 6.4% perceived their weight status as too thin, 74.8% as about right, and 18.9% as fat or overweight. On the basis of parental responses, 6.3% perceived their child’s weight status as underweight, 74.4% as about right, and 19.3% as overweight. When the child’s response matched the parent’s response (ie, too thin vs underweight; about right vs about right; fat or overweight vs overweight), perceptions were concordant. In examining the extent of overall concordance between personal weight perception and parental weight perception, 82.4%, 71.2%, and 64.7% of children/adolescents and parents in healthy weight, overweight, and obese categories, respectively, shared the same responses on perceived weight status (Table 2).

**Table 2:** Concordance of Personal and Parental Weight Perception According to Measured Weight Status of Healthy Weight, Overweight, and Obese Children and Adolescents Aged 8 to15 Years in the United States(N = 2,613), 2007–2010^a^

Personal Weight Perception	Parental Weight Perception	Concordant Perception	Healthy Weight (n = 1,557)	Overweight (n = 461)	Obese (n = 595)
Too thin	Underweight	Yes (1)	3.6	0.0	0.0
Too thin	About right	No	6.0	0.7	0.2
Too thin	Overweight	No	0.1	0.1	0.8
About right	Underweight	No	6.3	0.4	0.0
About right	About right	Yes (2)	78.3	61.7	16.4
About right	Overweight	No	1.2	11.4	24.4
Fat/overweight	Underweight	No	0.0	0.0	0.1
Fat/overweight	About right	No	4.0	16.2	9.9
Fat/overweight	Overweight	Yes (3)	0.5	9.5	48.3
Overall concordance		(1)+ (2)+ (3)	82.4	71.2	64.7

a Values are expressed as weighted percentages.

### Personal and parental weight misperception

In comparing differences between personal weight perception and measured weight status, 69.9% of children and adolescents perceived their weight status accurately, 27.3% underestimated, and 2.8% overestimated their measured weight status. In comparing differences between parental weight perception and measured weight status of the child, 73.7% of the parents perceived their child’s weight status accurately, 25.2% underestimated and 1.1% overestimated their child’s weight status.

In examining the accuracy rates of personal and parental weight perception within each BMI group, both children and adolescents (85.9%) and parents (88.3%) showed the highest accuracy rate when the child was in the healthy weight category (Figure). However, only about one-fourth of children and adolescents and one-fifth of parents were able to accurately identify the child’s weight status when the child was overweight. When the child was obese, parental weight perception had a higher accuracy rate (73.5%) than their child’s weight perception (58.3%).

**Figure:**
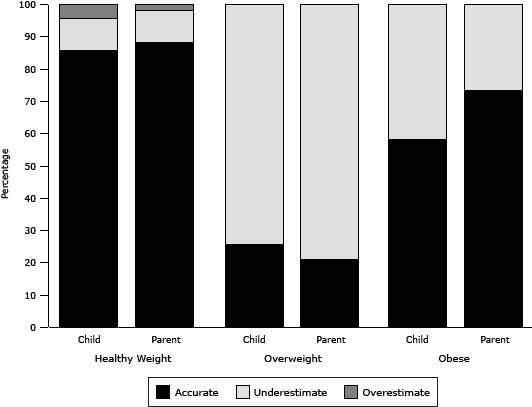
Accuracy of child’s and parent’s weight perception (weighted percentages) in healthy weight, overweight, and obese children and adolescents aged 8 to 15 years in the United States, National Health and Nutrition Surveys, 2007–2010. Both children and adolescents (85.9%) and parents (88.3%) showed the highest accuracy rate when the child was in the healthy weight category. Only 25.7% of children and adolescents and 21.0% of parents accurately identified the child’s weight status when the child was overweight. When the child was obese, parental weight perception had a higher accuracy rate (73.5%) than their child’s personal weight perception (58.3%). **Table:** 

Weight Category	Healthy Weight	Overweight	Obese
**Child’s perception of weight status, %^a^**			
Accurate	85.9	25.7	58.3
Underestimate	9.7	74.3	41.7
Overestimate	4.4	0	0
**Parent’s perception of child’s weight status, %**			
Accurate	88.3	21.0	73.5
Underestimate	9.9	79.0	26.5
Overestimate	1.8	0	0

^a^ Percentages are weighted %.

### Personal and parental weight misperception and self-reported attempted weight loss

The results of our multivariable-adjusted logistic regression models showed that the odds of self-reported attempted weight loss was nearly 10 times as high among healthy-weight children and adolescents who overestimated their actual weight status as among those who perceived their weight status accurately (reference group). The adjusted odds of self-reported attempted weight loss was 3.89 (95% CI, 2.37–6.41) and 2.91 (95% CI, 1.83–4.61) times as high for overweight and obese children and adolescents, respectively, who perceived their weight status accurately as among those who underestimated their actual weight status (reference group). Parental weight misperception, however, was not significantly associated with increased odds of self-reported attempted weight loss, irrespective of the child or adolescent’s BMI status (Table 3).

**Table 3:** Association Between Weight Misperception and Self-Reported Attempted Weight Loss by Healthy Weight, Overweight, and Obese Children and Adolescents Aged 8 to 15 Years in the United States (N = 2,613), 2007–2010^a^

Weight Misperception	Crude OR (95% CI)	Adjusted OR^b^ (95% CI)
**Healthy weight children and adolescents**		
Personal weight misperception		
Accurate	1 [Reference]	1 [Reference]
Underestimate	0.29 (0.12–0.71)	0.28 (0.11–0.70)
Overestimate	10.97 (4.81–25.02)	9.51 (3.83–23.64)
Parental weight misperception		
Accurate	1 [Reference]	1 [Reference]
Underestimate	0.08 (0.03–0.20)	0.12 (0.04–0.31)
Overestimate	1.70 (0.71–4.07)	0.75 (0.17–3.35)
**Overweight children and adolescents**		
Personal weight misperception		
Accurate	4.41 (2.71–7.20)	3.89 (2.37–6.41)
Underestimate	1 [Reference]	1 [Reference]
Parental weight misperception		
Accurate	2.42 (1.50–3.90)	1.75 (0.95–3.22)
Underestimate	1 [Reference]	1 [Reference]
**Obese children and adolescents**		
Personal weight misperception		
Accurate	3.05 (2.11–4.40)	2.91 (1.83–4.61)
Underestimate	1 [Reference]	1 [Reference]
Parental weight misperception		
Accurate	1.62 (1.07–2.47)	1.25 (0.75–2.07)
Underestimate	1 [Reference]	1 [Reference]

Abbreviations: OR, odds ratio; CI, confidence interval.

a Analyses are weighted.

b Further adjusted for child’s proxy-reported age, sex, race/ethnicity, health insurance status, and annual household income.

In our multivariable-adjusted analyses, there were significant sex differences in self-reported weight loss attempts in the healthy weight group, with boys being less likely to have self-reported attempted weight loss (OR, 0.59; 95% CI, 0.39–0.88). There were no sex differences in the other weight groups. There were no significant differences in the association between race/ethnicity and self-reported attempted weight loss in the healthy weight and obese groups. However, among the overweight group, Hispanic blacks (OR, 2.03; 95% CI, 1.07–3.86) and Hispanics (OR, 2.23; 95% CI, 1.16–4.30) were significantly more likely to have self-reported attempted weight loss than non-Hispanic whites. Our analyses did not reveal any significant interaction of personal or parental weight misperception with sex or race/ethnicity, or interaction between personal and parental weight misperception.

## Discussion

To the best of our knowledge, this was the first study to assess the effect of a child’s personal weight misperception on self-reported attempted weight loss while also taking parental misperceptions of the child’s weight status into account. In this nationally representative survey of children and adolescents aged 8 to 15 years and their parents, the proportion of personal and parental weight misperception was substantial, particularly among overweight children and adolescents and their parents. Accurate personal, but not parental, weight perception was positively associated with self-reported attempted weight loss among overweight and obese children and adolescents. However, healthy weight children and adolescents who overestimated their weight status made unnecessary weight loss attempts.

Although accurate personal perception of being either overweight or obese has been linked to greater motivation to engage in healthy weight-related behaviors (13), increasing evidence suggests that actual and perceived weight are often not in agreement, and this discordance is more common among overweight and obese people. Data from US (13) and Canadian (19) studies found that overweight and obese youth were more likely to misperceive their actual weight. Where prior studies often collapsed overweight and obese into a single category of overweight (13,20), our study showed that personal misperception occurred more often when the child was overweight rather than obese. Further research is needed to determine what factors drive children’s perceptions of their weight and how best to maintain an optimal weight profile during the early, formative years of childhood.

Parents tend to be most influential in a child’s life and typically play a critical role in determining what food is available to their child as well as shaping eating behaviors (21). However, parents’ actions involving the child’s weight depend on their awareness of the child’s actual weight status (22). The published literature has shown an important disconnect between a child’s actual weight and parental perception of their child’s weight (23). Our results suggest that parents often do not perceive their child’s weight status accurately and that this misperception occurs most frequently when the child is overweight.

Accurate awareness of weight status is one of the motivators in weight loss attempts. In childhood obesity research, parents’ misperception of their child’s elevated weight status has been shown to be a barrier to their child’s participation in obesity prevention interventions (9). Data from the Youth Risk Behavior Surveillance System have shown that overweight adolescents in grades 9 through 12 with accurate weight perception were more likely to report engaging in positive weight-related behaviors than those who misperceived their weight status (13). An Australian study of adolescent students (grades 7–12) found that overweight and obese children who accurately perceived their weight status were more likely to try to lose weight and to be more physically active than those who misperceived their weight status (24). These prior studies, contrary to our study, did not examine the effect of a child’s personal weight perception while also taking parental weight perception into account. Our findings suggest that a child’s accurate weight perception was one of the major driving forces behind taking action to lose weight. In addition, although the child’s personal weight perception agreed with parental weight perception only 77% of the time, we did not observe a significant interaction between personal and parental weight misperception. These findings should be interpreted with appropriate caution, however, since we did not know if these children and adolescents actually engaged in weight loss activity as they reported and if they did so voluntarily.

Our findings indicate that unnecessary weight loss attempts occurred among healthy weight children and adolescents who overestimated their weight status. Although appropriate weight loss can decrease the risk for chronic disease in adulthood, overemphasis on thinness in childhood and adolescence may lead to unhealthy weight-loss practices and misperceptions (25). An ongoing concern remains that the preference for thinness in the mass media may have a negative influence in shaping children’s and adolescents’ concept of their ideal body image (25). Because NHANES does not collect information about the child’s body image, we were unable to examine the potentially important role of this factor in the present findings; however, several studies have shown that altered body image is associated with eating disorders, ineffective dieting, and low self-esteem among children and adolescents (26–28). Future studies are needed to determine why normal-weight children perceive themselves as being overweight.

Although it is unclear what factors may cause misperception of actual weight status, a prior study suggested that children may develop false perceptions of normal-weight status when they are frequently exposed to overweight and obese people in their home and school environments (19). A recent review suggested that parental weight misperception may be related to cultural beliefs about body size, lack of knowledge of the true definition of overweight, or unwillingness to accept that their child is overweight (29). Future studies examining factors contributing to weight misperception are needed to develop effective interventions to help children and parents accurately assess weight status. In addition, among children who accurately self-identified as being overweight, not all were actively engaged in weight loss, suggesting that weight misperception is only one of the contributors to weight control.

The strengths of this study include its population-based design and its contemporary sample of nationally representative children and adolescents. In addition, weight status based on measured height and weight has reduced the potential for reporting bias compared with studies that use self-reported data (30). Several limitations need to be acknowledged, however, when interpreting the present findings. First, NHANES data are cross-sectional, and our ability to infer causal pathways between weight misperception and attempted weight loss was limited. Second, we could not control for unmeasured, potentially confounding factors, such as child’s pubertal stage and psychosocial factors; siblings’ weight status; parent’s sex, BMI, and education level; and family history of obesity because this information was not captured in NHANES. Third, because measured weight status was based on the cutpoints of BMI percentiles, the misperception may relate to the child’s or parent’s inability to accurately assess the quantitative cutpoints. Fourth, although our findings indicate that accurate parental weight perception is not significantly associated with attempted weight loss reported by children and adolescents, parents were not directly asked about what they were doing about their child’s weight. Finally, attempted weight loss by children and adolescents was self-reported, which may be overreported because of social desirability bias. Future research on the validity of this measure is needed. In addition, participants who were trying to lose weight may not have been successful. This issue was not sufficiently captured in the NHANES data. Future longitudinal studies examining the effect of weight misperception on attempted weight loss and objectively measuring the success of weight loss are needed.

Our findings have important implications for childhood obesity prevention. Behavioral interventions for accurate weight perception are needed for children and parents, because intervention efforts related to weight loss may be ineffective if individuals do not recognize or acknowledge that they are overweight. Our findings indicate that one of the major driving forces behind taking action to lose weight comes from the child’s perception, which suggests the importance of intervening with the child directly. Health campaigns promoting weight loss among children who are overweight or obese and promoting healthy weight maintenance among children with a normal BMI need to take this notion into consideration.

## Figures and Tables

**Table:** 

**1. The activity supported the learning objectives.**				
**Strongly Disagree**				**Strongly Agree**
1	2	3	4	5
**2. The material was organized clearly for learning to occur.**				
**Strongly Disagree**				**Strongly Agree**
1	2	3	4	5
**3. The content learned from this activity will impact my practice.**				
**Strongly Disagree**				**Strongly Agree**
1	2	3	4	5
**4. The activity was presented objectively and free of commercial bias.**				
**Strongly Disagree**				**Strongly Agree**
1	2	3	4	5

## Post-Test Questions

### Article Title: Personal and Parental Weight Misperception and Self-Reported Attempted Weight Loss in US Children and Adolescents, National Health and Nutrition Examination Survey, 2007–2008 and 2009–2010

**CME Questions**

1. You are seeing a 12-year-old girl for an annual well-child examination. She states that she is "not fat." Her body mass index is in the 87th percentile for her age. What should you consider regarding national trends in pediatric obesity and habits to lose weight among participants in the current study?
  - The rate of obesity among children and adolescents has increased nearly 15-fold during the past 30 years
  - The rate of obesity has increased faster among children vs adolescents
  - In 2009-2010, nearly one-third of children and adolescents in the United States were obese
  - The rate of trying to lose weight in the current study was 6%
2. The parents of this patient are not concerned about her weight and believe that she falls into the healthy weight category. Which of the following statements regarding misperceptions of weight status in the current study is ***most*** accurate?
  - Most parents incorrectly believed that their children were overweight or obese
  - Children and adolescents tended to incorrectly view themselves as overweight or obese, whereas parents were more likely to incorrectly believe that their children were underweight
  - Underestimation rather than overestimation of weight status was the most common error among children/adolescents and parents
  - The weight status category associated with the highest degree of correct child/adolescent and parent perceptions was underweight
3. You inquire about weight loss interventions, and the patient reports that she hasn’t tried to diet or increase exercise. What should you consider regarding weight loss behaviors in the current study?
  - Perceived weight status had no effect on weight loss behaviors
  - The relationship was strongest between weight loss behaviors and persons who incorrectly overestimated their weight status
  - The relationship was strongest between weight loss behaviors and persons who correctly identified their weight status
  - Only children and adolescents who incorrectly considered themselves overweight or obese have substantial attempts at weight loss
4. Which of the following other variables was ***most*** significantly associated with more weight loss attempts in the current study?
  - Female gender
  - White race
  - Younger age
  - Parental misperception of overweight and obesity

**Evaluation**
